# Membrane Proteocomplexome of *Campylobacter jejuni* Using 2-D Blue Native/SDS-PAGE Combined to Bioinformatics Analysis

**DOI:** 10.3389/fmicb.2020.530906

**Published:** 2020-11-19

**Authors:** Alizée Guérin, Sheiam Sulaeman, Laurent Coquet, Armelle Ménard, Frédérique Barloy-Hubler, Emmanuelle Dé, Odile Tresse

**Affiliations:** ^1^UMR 1014 Sacelim, INRAE, Oniris, Nantes, France; ^2^UMR 6270 Laboratoire Polymères Biopolymères Surfaces, UNIROUEN, INSA Rouen, CNRS, Normandie Université, Rouen, France; ^3^UNIROUEN, Plateforme PISSARO, IRIB, Normandie Université, Mont-Saint-Aignan, France; ^4^INSERM, UMR 1053 Bordeaux Research in Translational Oncology, BaRITOn, Bordeaux, France; ^5^UMR 6290, CNRS, Institut de Génétique et Développement de Rennes, University of Rennes, Rennes, France

**Keywords:** foodborne pathogen, proteomics, functional genomics, complexes, membrane proteins, efflux pumps, regulation, blue native electrophoresis

## Abstract

*Campylobacter* is the leading cause of the human bacterial foodborne infections in the developed countries. The perception cues from biotic or abiotic environments by the bacteria are often related to bacterial surface and membrane proteins that mediate the cellular response for the adaptation of *Campylobacter jejuni* to the environment. These proteins function rarely as a unique entity, they are often organized in functional complexes. In *C. jejuni*, these complexes are not fully identified and some of them remain unknown. To identify putative functional multi-subunit entities at the membrane subproteome level of *C. jejuni*, a holistic non *a priori* method was addressed using two-dimensional blue native/Sodium dodecyl sulfate (SDS) polyacrylamide gel electrophoresis (PAGE) in strain *C. jejuni* 81–176. Couples of acrylamide gradient/migration-time, membrane detergent concentration and hand-made strips were optimized to obtain reproducible extraction and separation of intact membrane protein complexes (MPCs). The MPCs were subsequently denatured using SDS-PAGE and each spot from each MPCs was identified by mass spectrometry. Altogether, 21 MPCs could be detected including multi homo-oligomeric and multi hetero-oligomeric complexes distributed in both inner and outer membranes. The function, the conservation and the regulation of the MPCs across *C. jejuni* strains were inspected by functional and genomic comparison analyses. In this study, relatedness between subunits of two efflux pumps, CmeABC and MacABputC was observed. In addition, a consensus sequence CosR-binding box in promoter regions of MacABputC was present in *C. jejuni* but not in *Campylobacter coli*. The MPCs identified in *C. jejuni* 81–176 membrane are involved in protein folding, molecule trafficking, oxidative phosphorylation, membrane structuration, peptidoglycan biosynthesis, motility and chemotaxis, stress signaling, efflux pumps and virulence.

## Introduction

*Campylobacter* is a Gram-negative spiral-shaped bacterium. It has emerged as the leading cause of foodborne bacterial gastroenteritis in humans (Epps et al., 2013; Kaakoush et al., 2015; EFSA and ECDC, 2018). The number of campylobacteriosis cases has been increasing in Europe since 2005 and has reached an incidence of 65 per 100,000 people with 246–158 confirmed cases in 2018 (EFSA and ECDC, 2018). Most cases were attributed to *Campylobacter jejuni*, an invasive microorganism causing gastroenteritis associated with fever and frequent watery bloody diarrhea, abdominal pains and occasionally nausea (Moore et al., 2005; Silva et al., 2011; Epps et al., 2013). It is also associated with post-infection complications including the immune-mediated neurological disease Guillain-Barré Syndrome (Nachamkin, 2002; Alshekhlee et al., 2008), its variant Miller Fisher Syndrome (Ang et al., 2001) or reactive arthritis (Altekruse et al., 1999). Notably, the infectious dose is considered to be lower than the one for other foodborne pathogens as only 500–800 bacteria trigger human infection (Robinson, 1981; Black et al., 1988; Boyanova et al., 2004; Castano-Rodriguez et al., 2015). *Campylobacter* cost of illness was estimated at 2.4 billion euros per year in Europe (EFSA, 2016). *C. jejuni* infections are mainly associated with consumption of poultry and cross-contamination from poultry products (Hue et al., 2010; Guyard-Nicodeme et al., 2013; Hald et al., 2016). For the first time, the European Commission regulation has amended the regulation (EC) No 2073/2005 in 2017 on the hygiene of foodstuffs as regards *Campylobacter* on broiler carcasses stating a limit of 1000 CFU/g applied from January 2018. This microaerophilic, capnophilic and thermophilic microorganism requires fastidious growth conditions and it growth is rapidly hampered by several environmental stress conditions. Optimal growth is obtained using a modified atmosphere limited in dioxygen and enriched in carbon dioxide, a temperature between 37 and 45 C and a pH between 6.5 and 7.5 (Mace et al., 2015). Nonetheless, *C. jejuni* is able to survive harmful conditions by developing adaptation mechanisms in response to stress conditions throughout the food chain (Atack and Kelly, 2009; Rodrigues et al., 2016). Living as biofilms is also a phenotypical feature that was demonstrated for *C. jejuni*, indicating multiple surviving ways outside hosts (Turonova et al., 2015).

Proteomic techniques have been applied to *Campylobacter* to better understand how changes in genetic expression, bacterial state, nutrient limitation, food plant processing and environmental conditions could affect *C. jejuni* at the protein level (Tresse, 2017). Natural compartmentalization has facilitated subfraction proteome analyses of *Campylobacter* such as the cytosolic proteome (Kalmokoff et al., 2006; Bieche et al., 2012; Asakura et al., 2016), the membrane proteome (Seal et al., 2007; Cordwell et al., 2008; Scott et al., 2014; Watson et al., 2014), the inner or outer membrane proteome (Sulaeman et al., 2012) and the exoproteome (Kaakoush et al., 2010). In addition, the genomic and computational era have brought exciting and challenging prospects for proteomics like assigning a function to each protein and subsequently its relationship to other proteins in the cell. Functional genomics and protein structural modeling approaches can predict protein-protein interactions (PPIs), which constitutes the theoretical protein interactome of an organism. Predicted interactomes, including potential stable or transient PPIs, are limited to databases content but PPIs already demonstrated to be biologically functional, specific genetic organizations (operons, gene clusters and regulons) or structural features (domains and loops) (Planas-Iglesias et al., 2013; Wetie et al., 2013). Genomic analyses of the main pathogenic species of *Campylobacter*, revealed a lack of some of the well-described organizations into operons or gene clusters in Gram-negative bacteria (Parkhill et al., 2000). For instance, genes involved in the amino-acid biosynthesis are scattered in distinct loci across the genome of *C. jejuni* whereas they are organized into operons in other bacteria. In *H. pylori*, the closest specie relative to *Campylobacter*, the presence of some genetic elements organized into operons, gene clusters or islands could have contributed to the specialization of this pathogen (Sohn and Lee, 2011; You et al., 2012). In *C. jejuni*, the virulence variation among strains could not be assigned to any specific genetic organization other than point mutations in the virulence-associated genes or indels in individual loci (Bell et al., 2013). A reduced genetic organization has probably participated to the idiosyncrasy of *C. jejuni*.

The alternative method to identify PPIs, which does not result necessary from a specific genetic organization, is to detect complexes of proteins using non-hypothesis driven methods. When these complexes are composed of only protein subunits, the global approach is called proteocomplexomic. This is the case of the two-dimensional (2-D) blue native (BN)/SDS-PAGE which aims at highlighting intact protein complexes using mild non-ionic and non-denaturing detergents (Dresler et al., 2011; Lasserre and Menard, 2012; Wohlbrand et al., 2016). This method consists in separating native protein complexes according to their molecular mass during the first dimension and subsequently in separating protein subunits of each complex in SDS-denaturated conditions in an orthogonal second dimension. It has been applied with success to monitor oligomeric state, stoichiometry and protein subunit composition of protein complexes.

This study aimed at exploring protein machineries of *C. jejuni* at the membrane level. The bacterial membrane as a hydrophobic lipid structure is a suitable site for protein complex organization. Numerous well-characterized proteins embedded in the membrane are organized into functional units involved in various cellular processes. These membrane protein complexes (MPCs) could be also influenced by the membrane structural integrity and their molecular environment (Sachs and Engelman, 2006). In *didermata* such as *C. jejuni*, MPCs could be either organized throughout both membranes and the periplasmic space or specifically in the inner or in the outer membrane. The first objective was to apply and to optimize 2-D BN/SDS-PAGE technique on the *C. jejuni* membrane proteins to obtain reproducible gels. The second goal was to identify MPCs present in *C. jejuni* during optimal growth. As this analysis was conducted on the membrane compartment, it was called membrane proteocomplexomic analysis.

## Materials and Methods

### Bacterial Cell Cultures and Sample Preparation

The virulent *C. jejuni* strain 81–176 (NC_008787), whose whole genome is available in Genoscope Platform (MicroScope Vallenet et al., 2017), was selected for the experiments. A loopful of frozen 81–176 cells culture, conserved at −80°C in Brain-Heart Infusion (BHI) broth (Biokar, Beauvais, France) containing 20% sterile glycerol, was cultured on fresh Karmali agar plates (Oxoid, Dardilly, France) (Air Liquid, Paris, France) at 42°C for 48 h in microaerobic conditions (MAC) generated using gas replacement jars operated by MACSmics gassing system (BioMérieux, France) with a gas blend composed of 5% O_2_, 10% CO_2_, and 85% N_2_ (Air Liquid, Paris, France) and 4 filled/flushed cycles at −50 kPa as described in Mace et al. (2015). Cultures were obtained by inoculating 500 mL of BHI broth in a 1-L flask and incubating them for 16 h under MAC at 42°C in a rotary shaker.

### Membrane Protein Complex (MPC) Extraction

The cells were harvested by centrifugation for 20 min at 4°C at 6,000 × *g*. The supernatant was discarded and about 3 g of dry pellet was obtained. The cells were washed twice with lysis buffer containing 50 mM Tris, 750 mM 6-amino-n-caproic acid as a zwitterionic salt, with each wash followed by centrifugation at 6,000 × *g* for 20 min at 4°C. The cells were then resuspended in 5.5 mL of lysis buffer supplemented with 60 μL phenylmethylsulfonyl fluoride (PMSF) and sonicated at 50 kHz for 6 × 30 s with 5 min intervals on ice (Vibracell 72434, Bioblock Scientific, Illkirch, France) as previously described by Bieche et al. (2012). The proteins present in the supernatant were then collected and centrifuged twice at 10,000 × *g* for 30 min at 4°C in order to remove the cellular debris. The whole protein lysate was treated with 0.2 mg/ml DNase I for 1 h at 25°C and then ultra centrifuged at 100,000 × *g* for 1 h at 4°C. The pellet containing membrane complexes was resuspended in 10 mL of lysis buffer with 50 μL PMSF supplemented with the mild detergent Dodecyl-β-D-Maltoside (DDM) (Sigma, France) at concentrations ranging from 1 to 5% (w/v) to maintain the integrity of protein complexes and limiting dissociation or denaturation as previously recommended by Bernarde et al. (2010). After 15 min on ice, each sample solubilized with DDM was directly ultra-centrifuged at 100,000 × *g* for 1 h at 4°C. The MPC extraction was performed in triplicate from three independent cultures. Aliquots of the supernatant containing the membrane protein complexes were stored at −80°C. The protein concentration of membrane complexes was determined using the Micro BCA^TM^ Protein Assay Kit (Perbio Science, Brebieres, France) according to the manufacturer protocol.

### MPC Separation Using 2-D BN/SDS-PAGE

#### First Dimension in Native Conditions (BN-PAGE)

The first dimension was performed in a blue native polyacrylamide gel (BN-PAGE) according to Schagger (Schagger and von Jagow, 1991) with the following modifications. The MCP separation using BN-PAGE gels (15 cm × 16 cm × 0.1 cm) was assayed on linear acrylamide gradients: 4–14% (w/v), 4–18% (w/v), 8–18% (w/v) or 10–20% (w/v) using a gradient forming unit and Protean II cell (Bio-Rad, Hercules, CA, United States). Each separating gel was overlaid with a 3% stacking BN-PAGE. Both anode and cathode buffers contained 50 mM Tris and 75 mM Glycine. Only the cathode buffer was supplemented with 0.002% (w/v) Coomassie Blue G250 (Serva Biochemicals, Heidelberg, Germany). The assembly of gels were embedded with anode and cathode buffers and maintained at 4°C for 3 h before loading the protein sample. A volume of 1–5 μL of sample buffer (500 mM 6-amino-*n*-caproic acid and 5% Serva blue G) was added to DDM-solubilized membrane protein complex samples. Thyroglobulin (669 kDa), ferritin (440 kDa), catalase (232 kDa), lactate dehydrogenase (140 kDa) and BSA (67 kDa) were used as high molecular weight native protein marker mixture (GE Healthcare, Buckinghamshire, United Kingdom). The migration was run at 4°C with 1 W per gel and limited at 150 V and 90 mA during 4 to 48 h according to the assays.

To check the optimal solubilization of the protein complexes using DDM, migration through BN-PAGE was performed as described above with 3% stacking gel. Samples of 10 or 20 μg of protein complexes solubilized in 1, 2 or 5% (w/v) DDM were prepared as described above and loaded for each lane of the BN-PAGE. For protein complex analyses, gels were silver stained and scanned with a GS-800 densitometer (Bio-Rad) operated with the Quantity One^®^ software (Bio-Rad) at the resolution of 42.3 microns as described previously by Sulaeman et al. (2012). For the protein identification, the gels were loaded with 50 μg of protein complexes. Following the 1-D migration, the protein complexes in the BN-PAGE were fixed using the kit Bio Safe^TM^ Coomassie G-250 Stain (Bio-Rad) according to the manufacturer’s instructions.

#### Second-Dimension SDS-PAGE

The second dimension was performed under denaturing conditions using 10% (w/v) acrylamide SDS-PAGE (15 cm × 16 cm × 0.15 cm). An individual lane was cut off from the first dimension BN-PAGE using a glass plate. Gel lane was equilibrated for 5 min in a buffer containing 1% (w/v) SDS and 125 mM Tris. Then, the proteins were reduced for 15 min into equilibrating buffer supplemented with 50 mM dithiothreitol (DTT) (Sigma, France), and subsequently alkylated for 15 min in equilibrating buffer supplemented with 125 mM iodoacetamide (Bio-Rad). An ultimate washing step lasting 5 min was performed in the equilibrating buffer without supplement. After polymerization of the separating SDS-PAGE and equilibration, the gel lane was laid on a plastic support and introduced between the gel glass plates over the separation gel and embedded with low-melting agarose. Migration was carried out for 4 h at 16°C at 300 V maximum and 10 mA/gel for the first 45 min and then at 20 mA/gel. After migration, proteins were silver stained and scanned as described above.

### In-Gel Trypsin Digestion

The silver-stained spots separated by SDS-PAGE were excised manually. At first, the spots were discolored, then washed and reduced/alkylated using an automated system (MultiProbe II, Perkin Elmer, France) as following: each spot was washed several times in water, once in 25 mM ammonium carbonate and dehydrated with acetonitrile (ACN). After drying the gel pieces, the reduction was achieved by incubation for 1 h with 10 mM DTT at 55°C. The alkylation was achieved by incubation the samples with 25 mM iodoacetamide for 1 h at room temperature. Finally, the gel spots were washed three times in water for 10 min, again alternating between ammonium carbonate and ACN. The gel pieces were completely dried before trypsin digestion and rehydrated by trypsin addition. The digestion was carried out overnight at 37°C. The gel fragments were subsequently incubated twice for 15 min in a H_2_O/ACN solution and in ACN to allow extraction of peptides from the gel pieces. The peptide extracts were then pooled, dried and dissolved in 10 μL starting buffer for chromatographic elution, consisting of 3% (v/v) ACN and 0.1% (v/v) formic acid in water.

### Protein Identification by LC MS/MS

The peptides were enriched and separated using a lab-on-a-chip technology (Agilent, Massy, France) and fragmented using an on-line XCT mass spectrometer (Agilent). The fragmentation data were interpreted using the Data Analysis program (version 3.4, Bruker Daltonic, Billerica, MA, United States). For the protein identification, the MS/MS peak lists were extracted, converted into mgf-format files and compared to the *C. jejuni*, strain 81–176 protein database (UniprotKB, CP000538 for the chromosome, CP000549 for plasmid pTet and CP000550 for plasmid pVir) with the MASCOT Daemon search engine (version 2.6.0; Matrix Science, London, United Kingdom). The following search parameters were used: trypsin was used as the cutting enzyme, the mass tolerance for monoisotopic peptide window was set to ±1.0 Da and the MS/MS tolerance window was set to ±0.5 Da. Two missed cleavages were allowed. Carbamidomethylation, oxidized methionine, acetylation and pyroglutamate in Nt and amidation in Ct were chosen as variable modifications. Generally, the peptides with individual ions scores higher than the score indicated for *p* < 0.05 were selected. The proteins with two or more unique peptides matching the protein sequence were automatically considered as a positive identification. The main raw data are presented in Supplementary Data Sheet S1. Other raw data are available upon request.

### Western Blotting

The western blots of 2-D BN/SDS PAGE were performed according to Sulaeman et al. (2012). Briefly, prior to transfer, the 2-D SDS gels were cut into two horizontal sections and each section was soaked for 15 min in transfer buffer. Then, the proteins of each gel section were transferred to a nitrocellulose membrane by electrophoresis using Mini Trans Blot (Bio-Rad). The transferred proteins were then probed with a 1/2000 dilution of antibody anti-PorA or antibody anti-CadF. The immunoreactive proteins were detected using a 1/2000 dilution goat-anti-rabbit alkaline phosphatase antibody [Anti-Rabbit IgG, F(avb)2 fragment-Alkaline Phosphatase, Sigma, Saint-Quentin-Fallavier, France], followed by BCIP/NBT staining (Bio-Rad). Gels were scanned using the GS-800 Imaging densitometer (Bio-Rad).

### Bioinformatic Analyses

#### *In silico* Determination of Complex Function

The presence and organization of genes encoded protein subunits of identified complexes in 81–176 and other *Campylobacter* complete genomes was explored using the Platform MicroScope described in Vallenet et al., 2017. The biological function of these proteins and complexes were inferred using KEGG and Microcyc (Kanehisa et al., 2016). The conformation analyses of the proteins alone or in a complex were performed using UniProt (Zommiti et al., 2016), RCSB (Stephen et al., 2018), RCSB PDB (Young et al., 2018), Swiss-model (Biasini et al., 2014) and OPM (Lomize et al., 2012). The functional links between partners of complex was explored using STRING (Szklarczyk et al., 2017). If the complex protein subunits were not identified in *Campylobacter*, homologous genes were searched using conventional gene alignment tools (BlatN).

#### Phylogenetic Tree of Efflux Pumps

The protein sequences of CmeA, CmeB, CmeC and MacA, MacB, putMacC (for putative MacC) on fourteen complete genomes of *C. jejuni* strains and six complete genomes of *Campylobacter coli* strains were recovered (Supplementary Data Sheet S1). MAFFT alignments (reference) and Fast Tree (reference) phylogenetic trees were performed using Geneious R9 and visualized using FigTree V1.4.3 software^1^. The proteins structures predictions were determined using Philus transmembrane prediction server (reference,)^2^.

#### Distribution of Efflux Pumps CmeABC and MacABputC in Bacteria

BlastP analyses were performed for each protein sequence of the components of these efflux pumps in *C. jejuni* 81–176 against domain bacteria in RefSeq (NCBI Reference Sequence Database) protein database. The general parameters applied for similarity validation were Max target sequences at 20000, automatically adjust parameters for short input sequences, expect threshold at 10, word size at 6 and max matches in a query range at 0. Scoring parameters were obtained from the blosum62 matrix, gap costs with existence at 11 and extension at one with a conditional compositional score matrix adjustment.

#### Identification of CosR DNA-Binding Box

The sequence logo of CosR-binding box previously defined by Turonova et al. (2017) was used to check the presence of cosR-binding box in the promoter regions of the operons encoding the efflux pumps *cme*ABC and *mac*ABputC among the complete genomes of *C. jejuni* and *C. coli* (Supplementary Data Sheet S1). For that, a sequence length of maximum 120 pb in the intergenic regions upstream to *cmeA* and *macA* was recovered. Based on these sequences, a new sequence logo of CosR DNA-binding box with sequences was drawn using WebLogo platform^3^ (Crooks et al., 2004).

## Results

### Optimization of Protein Complex Solubilization and Separation

**FIGURE 1:**
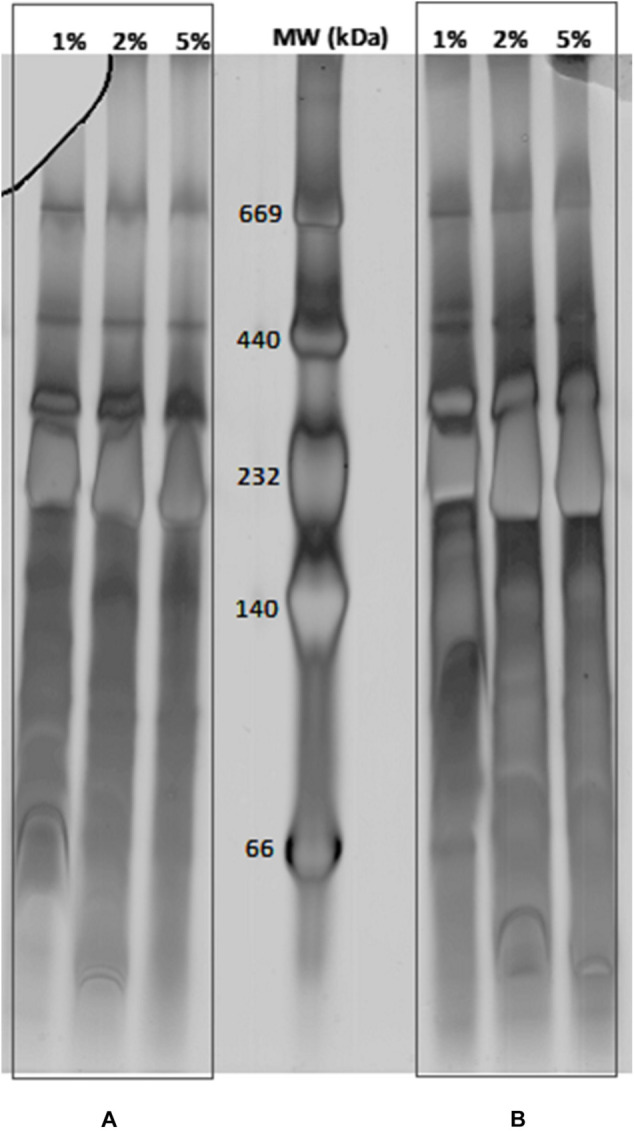
Separation of membrane protein complexes from *C. jejuni* Strain 81–176 by BN-PAGE. 10 μg **(A)** and 20 μg **(B)** of membrane protein complexes were solubilized with DDM at concentrations ranging from 1, 2, and 5% (w/v). Mass marker are indicated in the central lane (MW in kDa).

The BN-PAGE was applied first to check the solubilization efficiency of the extracted MPCs. In order to approach a useful detergent concentration for protein complex solubilization for BN-PAGE, Dodecyl-β-D-Maltoside (DDM) has turned out to be suitable (Schägger, 2002). The solubilization of the membrane was tested in a concentration of 1, 2, or 5% of DDM. The loading quantities of 10 μg or 20 μg of MPCs on BN-PAGE were found appropriate to determine the reliable concentration of DDM detergent for *C. jejuni* (Figure 1). In native conditions, MPCs migrate according to their mass and their form in space (steric hindrance). The bands detected in BN-PAGE in the range of 20 kDa to 670 kDa indicate that MCPs were stable after membrane solubilization and during the separation. All tested concentrations of DDM (1, 2, and 5%) seem to be suitable to solubilize MPCs from *C. jejuni*. However, over the three independent extractions, less reproducibility was obtained with 5% DDM for the lower molecular mass complexes (not shown). Consequently, only 2% DDM were selected to explore MPCs of *C. jejuni* 81–176. In addition, the better separation of MPCs was obtained by extending the migration time until 40 h (Supplementary Figure S1). Acrylamide gradients of BN-PAGE were also optimized by testing different couples of lower concentrations (ranging from 4 to 10%) and higher concentration (ranging from 14 to 20%). Even though some protein complexes were more distinct on some gradients, the linear gradient which gives more detectable complexes was 4 to 18% of acrylamide. In general, a lack of reproducibility of the 2-D gels and vertical smearing altering the resolution of MPCs subunits on SDS-PAGE were frequently reported. To circumvent these biases, many gels were run in previous studies until obtaining at least three similar replicates. In the present study, obtaining gel repeatability in a consecutive manner was the first goal. This goal was reached by decreasing vertical smearing, adjusting the thickness of the gels, fixing the supports and isolating the homemade strip from glass using a five-step protocol (Supplementary Figure S1). All these optimization steps resulted in performing consecutive reproducible gels. The reproducibility was validated once three consecutive profiles could be aligned with the same number of detected spots. The results of optimization steps during the first and second dimensions are presented in Supplementary Figure S2.

### Analysis of 2D-BN/SDS-PAGE Data

**FIGURE 2:**
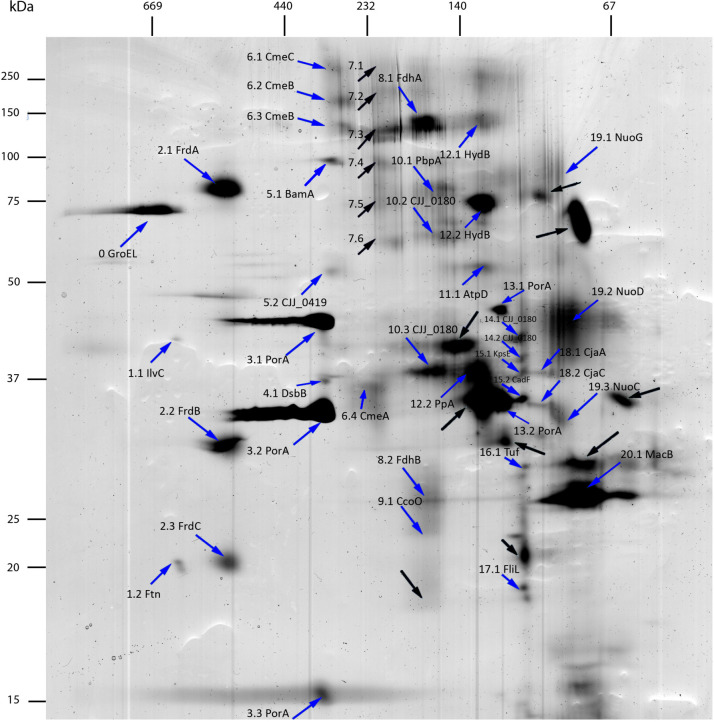
2D-BN/SDS-PAGE separation of membrane protein complexes of *C. jejuni* 81–176. Acrylamide gradient was 4–18% for BN-PAGE and acrylamide concentration was 10% for SDS-PAGE. Mass markers for BN-PAGE and SDS-PAGE are respectively, indicated at the top left side of the gel. Proteins identified by LC-MS/MS are indicated by blue arrows. Proteins that do not reach identification criteria after LC-MS/MS analysis are indicated with black arrows. First numbers correspond to complexes and second numbers to subunits of these complexes.

Using the criteria mentioned by Reisinger and Eichacker (2006), all protein spots that are located vertically below each other with a similar shape on the 2-D were considered as subunits of one MPC. Consequently, the complexes were numbered from the left side to the right side of the 2-D gel and the detected subunits for each complex with a second number starting from the top of the gel (Figure 2). The spots, which were located side by side in a horizontal row, could potentially be an identical subunit in protein complexes of different molecular masses. If we assume that the molecular mass of a protein complex is increased during assembly of its structural subunits, the analysis of the protein pattern allows to determine the stepwise subunit assembly. The lower toward the higher molecular mass should correspond to complexes located from the right side of the gel toward its left side (Reisinger and Eichacker, 2008). When a complex was composed of subunits from the same gene product, they were called multihomooligomeric complexes and when they were composed of different subunits, they were named multi hetero-oligomeric complexes as described before (Bernarde et al., 2010). Some complexes could not be detected due to a relatively low abundance in the membrane, solubilization parameters or separation parameters. In addition, depending on the solubilization and separation parameters more than one protein complex could run at the same molecular mass during BN-PAGE. This was observed more frequently for smaller protein complexes. In this case, the spot identification helps to separate different complexes when the biological function was previously described. The solubilization, the migration parameters, the interaction between subunits and the subunit organization in or associated with the membrane could also result in partial identification of complexes.

The identification of the spots was achieved by nanoLC MS/MS and validated using Mascot score (Table 1 and Supplementary Data Sheet S1). The western blots using polyclonal antibodies anti-PorA and anti-CadF were used to target specific outer membrane proteins (Supplementary Figure S3). Three spots of PorA and one spot of CadF could be identified using Western-blot confirming the identification performed by LC MS/MS. In addition, the LC MS/MS identified two supplementary spots of PorA indicating that it has a probable higher sensitivity than Western-blot according to the protein abundance. As expected, PorA, also named major outer membrane protein (MOMP) is among the predominant proteins. It was previously reported that PorA could account for 45% of the total visible membrane proteins of *C. jejuni* (Cordwell et al., 2008).

**TABLE 1:** Description of membrane protein complexes identified in *C. jejuni* strain 81–176 using two-dimensional BN/SDS-PAGE.

Complex function	Complex ID	Spot ID	Access No. (NCBI)	Protein ID	Gene name	Mascot score (a)	NPM/PC (%) (b)	pI/MW (kDa) (theoretical)
Oxidative phosphory-lation	2	2.1	EAQ73056.1			413	12/20	6.36/74
		2.2	EAQ73378.1	Fumarate reductase iron-sulfur subunit	*frdB*	153	4/22	5.37/27
		2.3	EAQ73136.1	Fumarate reductase cytochrome b-556 subunit	*frdC*	38	2*/5	9.37/30
	9	9.1	EAQ72593.1	Cytochrome c oxidase, cbb3-type, subunit II	*ccoO*	136	4/23	5.86/25
	11	11.1	EAQ71910.1	F0F1 ATP synthase subunit beta	*atpD*	102	2/5	4.97/51
	19	19.1	EAQ72569.1	NADH dehydrogenase subunit G	*nuoG*	205	6/9	5.49/93
		19.2	EAQ72908.1	NADH dehydrogenase subunit D	*nuoD*	121	3/7	5.51/47
		19.3	EAQ72659.1	NADH dehydrogenase subunit C	*nuoC*	131	2/10	7.77/31
Respiration	8	8.1	EAQ72956.1	Formate dehydrogenase, alpha unit, selenocysteine-containing	*fdhA*	401	12/19	6.09/83
		8.2	EAQ72781.1	Formate dehydrogenase, iron-sulfur subunit	*fdhB*	65	2*/9	5.99/24
	12	12.1	EAQ72716.1	Quinone-reactive Ni/Fe-hydrogenase, large subunit	*hydB*	98	2/6	6.26/64
		12.2	EAQ72716.1	Quinone-reactive Ni/Fe-hydrogenase, large subunit	*hydB*	164	6/14	6.26/64
	0	0	EAQ72817.1	Chaperonin GroEL	*groEL*	698	16/39	5.02/58
Protein	4	4.1	EAQ71919.1	DsbB family disulfide bond formation protein	*dsbB*	139	3/7	8.57/57
biosynthesis and	10	10.1	EAQ73315.1	Penicillin-binding protein 1A	*pbpA*	81	3/5	8.35/73
folding		10.2	EAQ73158.1	Methyl-accepting chemotaxis protein	*CJJ_0180*	101	3/5	4.94/73
		10.3	EAQ73158.1	Methyl-accepting chemotaxis protein	*CJJ_0180*	132	4/6	4.94/73
	3	3.1	EAQ72728.1	Major outer membrane protein	porA**	2004	14/49	4.72/46
Efflux pumps,		3.2	EAQ72728.1	Major outer membrane protein	porA**	1989	14/48	4.72/46
virulence and		3.3	EAQ72728.1	Major outer membrane protein	porA**	1133	8/30	4.72/46
molecules	5	5.1	EAQ73202.1	Outer membrane protein	*bamA*	70	2*/4	5.57/83
trafficking		5.2	EAQ72997.1	Conserved hypothetical protein (putative lipoprotein)	*CJJ_0419*	196	5/17	8.48/37
	6	6.1	EAQ73082.1	RND efflux system, outer membrane lipoprotein CmeC	*cmeC*	129	7/10	5.14/55
		6.2	EAQ73146.1	RND efflux system, inner membrane transporter CmeB	*cmeB*	101	2/2	6.48/114
		6.3	EAQ73146.1	RND efflux system, inner membrane transporter CmeB	*cmeB*	121	2/2	6.48/114
		6.4	EAQ72976.1	RND efflux system, membrane fusion protein CmeA	*cmeA*	102	3/10	8.29/40
	13	13.1	EAQ72728.1	Major outer membrane protein	porA**	231	4/11	4.72/46
		13.2	EAQ72728.1	Major outer membrane protein	porA**	419	10/30	4.72/46
	15	15.1	EAQ72952.1	Capsular polysaccharide ABC transporter	*kpsE*	136	3/8	6.22/43
		15.2	EAQ72738.1	Outer membrane fibronectin-binding protein	cadF**	55	2/8	5.89/36
	20	20.1	EAQ73027.1	Macrolide-specific efflux protein macB	*CJJ_0*636	35	2*/1	9.25/70
	18	18.1	EAQ72087.1	CjaA protein	*cjaA*	21	1/4	5.69/31
		18.2	EAQ72374.1	CjaC protein	*cjaC*	250	6/25	6.48/28
Mobility	17	17.1	EAQ72823.1	Flagellar basal body-associated protein FliL	*fliL*	47	1/15	4.93/20
Unknown	1	1.1	EAQ73148.1	Ketol acid reductoisomerase	*ilvC*	365	8/27	6.1/37
		1.2	EAQ72988.1	Non-heme iron-containing ferritin	*ftn*	73	2/16	5.34/20
	14	14.1	EAQ73158.1	Methyl-accepting chemotaxis protein	*CJJ_0180*	94	2/4	4.94/73
		14.2	EAQ73158.1	Methyl-accepting chemotaxis protein	*CJJ_0180*	111	4/6	4.94/73
Other	16	16.1	EAQ73030.1	Elongation factor Tu	*tuf*	60	2/8	5.11/44

None of the proteins from the complex no. 7 have been identified. (a) From all identified peptides (b) Peptides with only a significant individual ion score were considered for NPM (Number of Peptide Match) and PC (Protein Coverage). *One peptide with a non-significant score but mainly identified from y and b ions (see MS/MS fragmentation in Supplementary Data) are included to validate the identification. **Proteins verified by western blot.

### Protein Complex Identification by Two-Dimensional (2-D) Blue Native (BN)/SDS-PAGE

Overall, 55 spots were submitted to LC-MS/MS analysis (Figure 2 and Table 1). Among them, nine isolated spots and all spots of complex 7 could not be identified, although attempts using different MS technologies were performed. No contamination with exogenous protein, like keratin, was detected. The spectrograms seem to show noise-to-signal trouble shootings. Two proteins identified with a low scoring (Mascot score <30) were discarded from the analysis. The remaining spots could be grouped into 20 complexes according to their location in the gel, identification and biological functions when available. Overall, 39 proteins predicted as membrane proteins or membrane associated proteins were identified indicating the efficiency of the extraction of the protein complexes from *C. jejuni* 81–176. These complexes were grouped according to biological functions of KEGG classification (Table 1). Four complexes are involved in oxidative phosphorylation, two in the respiration process, seven in molecules trafficking, three in protein biosynthesis and folding, one in motility and three with unknown functions in the membrane. Altogether, 6 multi hetero-oligomeric and fourteen multihomooligomeric complexes were identified (Table 1). Certain identified complexes were already described in *C. jejuni* such as efflux pump CmeABC (Gibreel et al., 2007), validating this technique to identify MPC in this bacteria. However, novel complexes are presented, such as complexes 2 and 8 comprised of FrdABC and FdhAB, respectively. These complexes were already described in *Helicobacter pylori* by Bernarde et al. (2010) and *Eubacterium acidaminophilum* (Graentzdoerffer et al., 2003), respectively.

### *In silico* Analysis of Efflux Pumps CmeABC and MacAB

**FIGURE 3:**
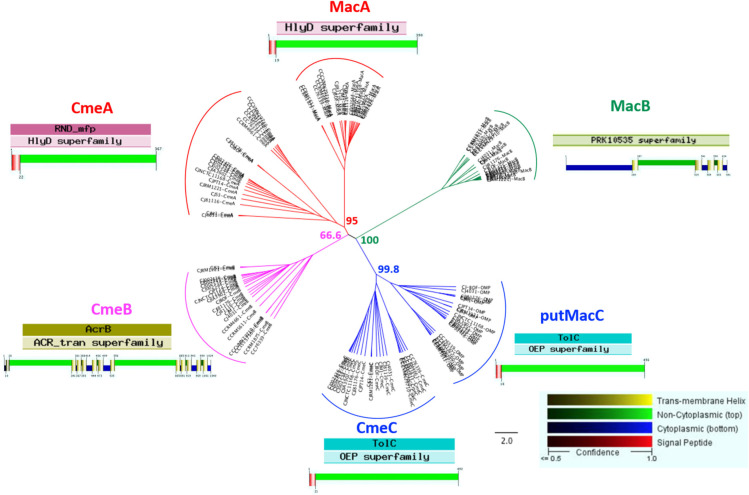
Phylogenetic tree of proteins belonging to efflux pumps CmeABC and MacABputC. Alignment was performed using MAFFT (Geneious R9) with functional domain and protein structure (Philus) in 14 *C. jejuni* strains and 6 *C. coli* strains. Bootstraps percentages were added to internal branches for 1000 replicates. Strains used in this study are listed in Supplementary Table S1.

Among the 20 identified complexes, subunits belonging to two efflux pumps were detected in complex 6 with CmeA, CmeB and CmeC and complex 20 with MacB (Figure 2 and Table 1). The subunits of these two pumps were further investigated in this study. CmeABC is a multridrug efflux system in *Campylobacter* working as an RND efflux pump (Lin et al., 2002; Akiba et al., 2006; Grinnage-Pulley and Zhang, 2015). It contributes to the resistance acquisition of *Campylobacter* to various antimicrobials including macrolides and fluoroquinolones (Yan et al., 2006; Gibreel et al., 2007; Jeon and Zhang, 2009). This efflux pump has also an important role in the resistance to bile (Lin et al., 2003). It includes the inner membrane drug transporter CmeB, the periplasmic membrane fusion protein CmeA and the outer membrane channel CmeC. These proteins can be glycosylated at various sites (Scott et al., 2011) which could explain two CmeB proteins identified with a different molecular weight (Figure 2). The bioinformatics analysis confirmed the organization in operon of the two efflux pumps amongst both *C. jejuni* and *C. coli*. For the other efflux pump, the subunit MacB, previously identified to be associated with MacA, was detected in the multihomooligomeric complex 20 which belongs to the efflux pump specific to macrolides (Yum et al., 2009; Bogomolnaya et al., 2013). Using Platform MicroScope, STRING, blastp and blastn, both DNA and protein sequences of these efflux pump partners were analyzed across *C. jejuni* and *C. coli*. Genes *cmeA* and *macA* are homologous and *cmeC* is homologous to a gene encoding a putative outer membrane protein (CJJ81176_0637) located downstream to *macB*. This putative outer membrane protein contains the same functional domain TolC as the one described in CmeC suggesting that this putative protein is probably the third partner of MacAB efflux pump. Further experimental assays will be required to validate the biological function of this putative protein for macrolide efflux pump operation. The phylogenetic and functional analyses confirm the similarities between proteins sequences of subunits of these two efflux pumps across *Campylobacter* strains (Figure 3 and Supplementary Table S1). CmeA and MacA belong to the HlyD superfamily showing a structure composed of the signal peptide and the non-cytoplasmic domain. The phylogenic analysis of the putative outer membrane protein CJJ81176_0637 confirmed it similarly to CmeC which likely is the third subunit of the efflux pump MacAB (Figure 3). Considering this data, this putative outer membrane protein was named putative MacC (putMacC) and the system MacABputC. In contrast to the similarly between sequences and structural functions of CmeC and putMacC in one hand and CmeA and MacA in the other hand, differences observed between CmeB and MacB is probably at the origin of the restriction of MacB to macrolides efflux.

### Analysis of the Potential Regulation of MacABC

**FIGURE 4:**
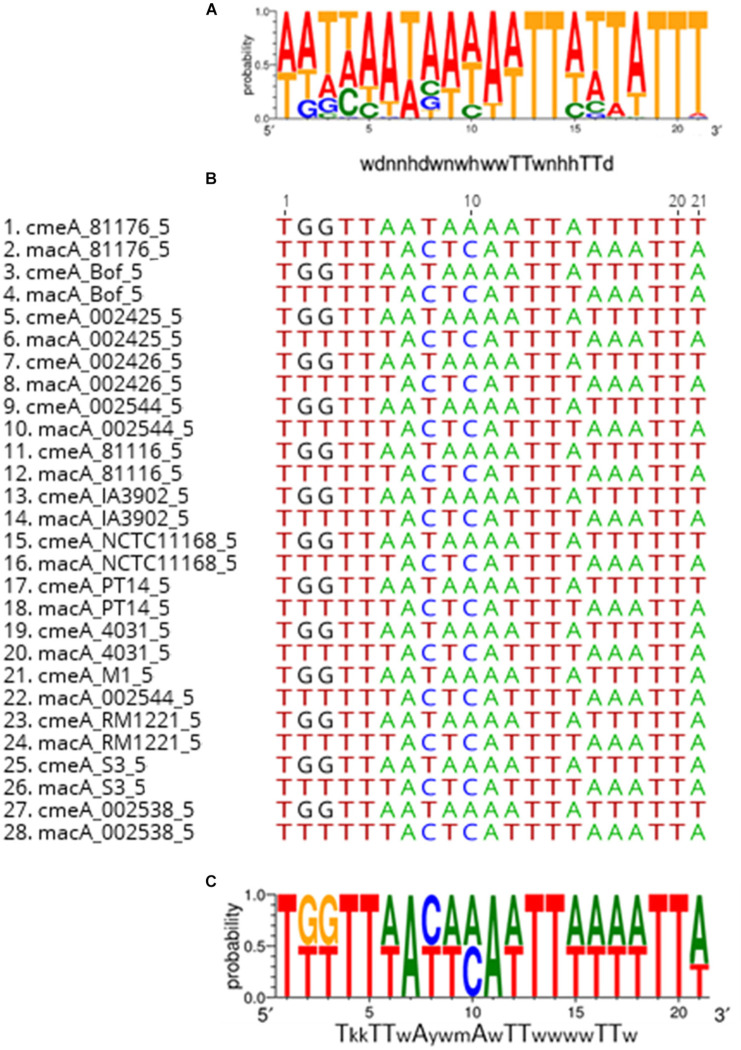
Consensus sequence logo of CosR-binding box of the upstream sequences of genes with CosR-binding capacity (*cme*A and *mac*A). Consensus sequence logo of CosR-binding box defined by Turonova et al. (2017) **(A)** alignment of upstream sequences of cmeA and macA by Geneious 9.1.8 **(B)** Consensus sequence logo of CosR-binding box redefined **(C)**. w-A or T; y-C or T; m-A or C and k-G or T.

The efflux pump CmeABC was previously shown to be regulated by CmeR and CosR (Lin et al., 2005; Hwang et al., 2012; Grinnage-Pulley et al., 2016) while no regulation was identified for MacABC. Using the CosR binding box sequence (5′-wdnnhdwnwhwwTTwnhhTTd- 3′) previously described by Turonova et al. (2017), *in silico* analysis revealed the presence of a CosR-like DNA-binding box upstream to *macA* in *C. jejuni* 81–176. Screening for the presence of the CosR binding box in the promoter region of *mac*A in the complete genomes of *C. jejuni* and *C. coli*, this consensus sequence was found in *C. jejuni* but not in *C. coli*. All the binding box DNA sequences of CosR in the *cme*A promoter region of both *C. jejuni* and *C. coli* strains and the *mac*A promoter region of *C. jejuni* strains were compared so as to propose a consensus sequence logo refined for CosR-binding box (Figure 4).

### Distribution of Gene Subunit Encoding CmeABC and MacABputC Across Bacteria Domain

**FIGURE 5:**
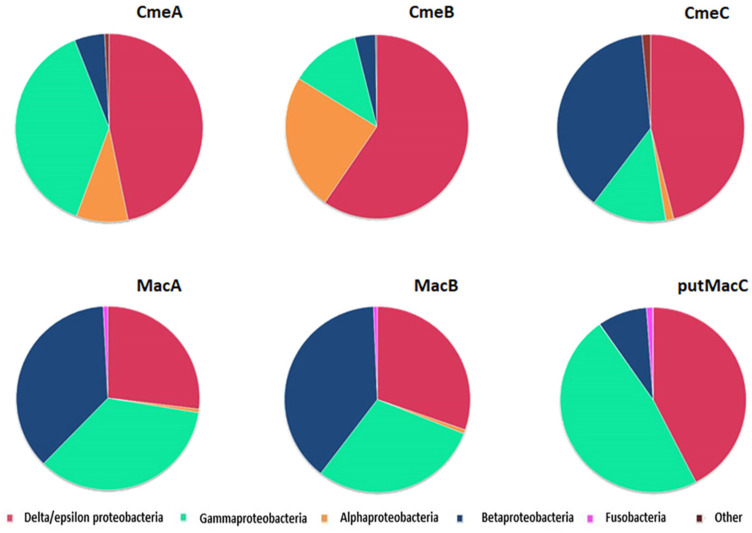
Distribution of CmeABC and MacABputC subunits in bacteria using Blastp with *C. jejuni* 81–176 protein sequences of each subunit as queries in RefSeq_protein database. The data are based on the hits presented in Supplementary Table S2.

Analyses of genes encoding proteins belonging to these two efflux pumps using across Bacteria domain Blastp analysis indicates that they are mainly observed in proteobacteria (Figure 5 and Supplementary Table S2). CmeA, CmeB, and CmeC were mainly found in the delta/epsilon proteobacteria groups while MacABputC also highly more represented in beta and gamma proteobacteria. Proteins of MacABputC were also found in the fusobacteriaceae family belonging to the fusobacterial phylum (Figure 5 and Supplementary Table S2). The presence of this third subunit in outer membrane (putMaC and CmeC) is probably crucial for the functionality of these complexes.

## Discussion

**FIGURE 6:**
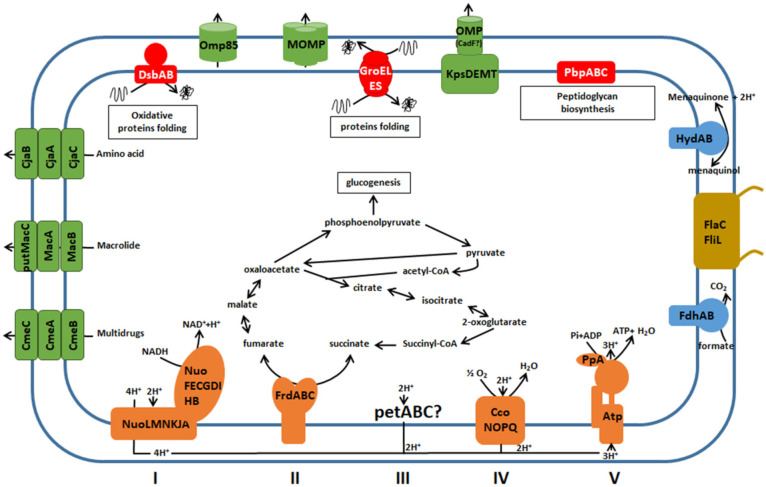
Membrane protein complexes identified by 2-D BN/SDS-PAGE in *C. jejuni* 81–176. Color of complexes corresponds to their function in cell: Green for molecules trafficking, Red for protein biosynthesis and folding, Blue for respiration, Yellow for motility, and Orange for the oxidative phosphorylation.

Beyond the protein mapping at the organism or a biological compartment scale using holistic approaches, identifying functional multi-subunit entities at the proteome level is a real challenge. Many cellular processes are carried out by sophisticated multi-subunit protein machineries, i.e., different protein complexes maintained by stable protein interactions. These functional entities could be defined as protein complexes composed of a minimal biologically structure of assembled protein subunits necessary for a specific cellular process (Reisinger and Eichacker, 2007). Membrane proteocomplexome of *C. jejuni* 81–176 cultivated in optimal growth conditions was explored using 2-D BN/SDS PAGE. The prerequisite goal was to obtain reproducible profiles after optimizing and stabilizing homemade strips. The objective was reached when three consecutive gels with resolved spots could be performed. Twenty-one MPCs were found with this method in *C. jejuni* 81–176 (Figure 6). We found incomplete complexes suggesting that subunits were probably lost during MPC extraction or unidentified by LC MS/MS.

### Oxidative Phosphorylation

Oxidative phosphorylation is the metabolic pathway in which bacteria use enzymatic complexes to re-oxidize cofactors and produce ATP. It ensures the electron transfer between electron donors and the final electron acceptor which is oxygen for aerobic and microaerobic bacteria such as *C. jejuni*. The redox reactions are carried out by a series of four protein complexes (I, II, III, and IV) located in the inner membrane. Membrane proteocomplexomic profiling revealed the presence of complexes involved in oxidative phosphorylation. Complex 19 corresponds to the NADH ubiquinone oxidoreductase, the first complex of the oxidative phosphorylation chain. This complex catalyzes the transfer of two electrons from NADH to quinone with the translocation of four protons across the inner membrane: NADH + H^+^ + Q + 4H^+^_*in*_ → NAD^+^ + QH_2_ + 4H^+^_*out*_ (Baranova et al., 2007; Weerakoon and Olson, 2008; Efremov and Sazanov, 2011; Baradaran et al., 2013). Genomic analysis revealed fourteen genes *nuo* organized in operon in *C. jejuni* and *C. coli* genomes with a highly conserved synteny. NuoFEGDCBI are involved in the hydrophilic domain of the NADH dehydrogenase complex while NuoAHJKLMN is localized into the membrane (Baradaran et al., 2013). The partners of this complex NuoC, NuoD and NuoG detected from our MPC fingerprinting are predicted to be localized on the basal part of the hydrophilic domain, close to the inner membrane. In *Escherichia coli*, NuoC and NuoD are fused and NuoG is close to them (Baranova et al., 2007; Baradaran et al., 2013). If these subunits are similarly organized in *C. jejuni*, this would indicate that 2% DMM MPC extraction, detection and subunit identification mainly selected this part of NADH dehydrogenase complex. The complex 2 corresponds to complex II of the oxidative phosphorylation chain. The fumarate reductase complex is generally composed of FrdA, FrdB and FrdC (Weingarten et al., 2009; Guccione et al., 2010; Jardim-Messeder et al., 2017). All three subunits were detected and identified on all the proteocomplexomic finger printings performed in the present study. In *C. jejuni*, this inner membrane system is bifunctional being able to catalyze both succinate oxidation and fumarate reduction (Guccione et al., 2010; Hofreuter, 2014). The succinate oxidation is favored under microaerobic conditions while the fumarate reduction is operated under oxygen-limited conditions. All three genes are close located on the genome of both *C. jejuni* and *C. coli*. FrdA is the fumarate reductase flavoprotein, FrdB is the iron-sulfur subunit and FrdC is the cytochrome B-556 subunit. This fumarate reductase complex FrdABC was also described in *H. pylori* (Pyndiah et al., 2007). A fourth partner, FrdD is described in *E. coli* (Rothery et al., 2005). However, the bioinformatic analyses did not reveal any homologous gene to FrdD in *C. jejuni* and *C. coli* genomes. This would indicate that complex 2 does exist and might be functional in *C. jejuni* and *H. pylori* without FrdD subunit. Any subunit of the ubiquinol-cytochrome c reductase, complex III of the oxidative phosphorylation, was not detected in this study. However, predicted functional partners of this proton pump were detected in *C. jejuni* genome: *petA* encoding the iron sulfur subunit, *petB* encoding the cytochrome b subunit and *petC* encoding the cytochrome c1 subunit which indicates the absence of this complex in optimal growth conditions or DMM limitations to extract all MPCs. Partners, function and pathways of cytochrome c oxidase (complex IV) in *Campylobacter* remain elusive. It is encoded by *ccoNOPQ*. The protein CcoO (Complex 9) was detected in our study. It corresponds to the subunit II of cytochrome c oxidase complex with a high affinity for O_2_ (Cosseau and Batut, 2004; Hofreuter, 2014). In complex V, the ATP synthase is usually composed of nine subunits, AtpABCDEFFGH often identified as subunit α, A, ε, β, C, B, B’, γ and δ (Rastogi and Girvin, 1999; Altendorf et al., 2000; Cingolani and Duncan, 2011; Okuno et al., 2011). The bioinformatics analyses revealed that genes *atpF, atpF’, atpH, atpA, atpG, atpD* and *atpC* are close located in *C. jejuni* and *C. coli* genomes while genes *atpB* and *atpE* are present in different loci. The proteocomplexomic analysis identified AptD (also called subunit β in complex 11). The membrane-bound ATP synthase is a key energy carrier in bacteria using the energy of an electrochemical ion gradient and the synthesis of ATP from ADP with inorganic phosphate (Rastogi and Girvin, 1999; Altendorf et al., 2000; Cingolani and Duncan, 2011; Okuno et al., 2011).

### Respiration

The complex formate dehydrogenase (complex 8) contributes to the respiration process by producing CO_2_ from formate oxidation (Hofreuter, 2014). This complex is composed of two FdhA and FdhB, two subunits detected on complexomic fingerprinting. As an asaccharolytic microorganism, carbon supply in *Campylobacter* is ensured from amino and organic acids and formate is one of the preferred substrate when hosted in poultry. The second detected complex involved the respiration process is hydrogenase complex HydAB complex 12. Two HydB with different weights (around 75 kDa and 130 kDa) were identified in complex 12 indicating the possible presence of a dimeric form of HydB (spot 12.1) in *C. jejuni* membrane. For these two complexes, single conserved copies of the encoding genes were observed in all analyzed genomes except for *C. jejuni* 4031 where a second copy of *FdhA* was found. Two other subunits for each complex (FdhC/FdhD and HydC/HydD) were previously identified in *C. jejuni* (Andreesen and Makdessi, 2008; Smart et al., 2009; Weerakoon et al., 2009; Pryjma et al., 2012; Shaw et al., 2012; Hofreuter, 2014). These two other partners interact with the main ones only under environmental stress conditions. The genes encoding these environment dependent conditions are present on *C. jejuni* and *C. coli* genomes. As MPCs of *C. jejuni* 81–176 were explored under optimal growth in our study, it is not a surprise not having detected them.

### Biosynthesis and Folding of Proteins

Several proteins are biosynthesized and folded by different complexes localized in membrane. Different membrane protein complexes were extracted and identified in this study.

The chaperonin GroEL (complex 0) is a cylindrical complex with two stacked heptameric rings with ATPase activity that binds non-native substrate protein (SP). GroEL is associated with cofactor GroES and form a nano-cage where SP can be folded up (Klancnik et al., 2006; Chi et al., 2015; Haldar et al., 2015; Motojima and Yoshida, 2015; Hayer-Hartl et al., 2016). As its cofactor GroES has a too small molecular weight (10 kDa), it could not be detected on our profiling fingerprinting. Another complex (complex 4) playing a role in protein folding was detected. DsbB-DsbA is a disulfide bond generation system operating in the oxidative pathway (Inaba et al., 2006; Inaba and Ito, 2008; Ito and Inaba, 2008; Grabowska et al., 2011; Sperling et al., 2013). In this study, only DsbB subunit was identified although two *dsb*A genes with 51% homology were present in *C. jejuni* 81–176 genome. DsbB localized in inner-membrane interacts with the periplasmic dithiol oxidase DsbA (Inaba et al., 2006; Inaba and Ito, 2008).

### Molecules Trafficking in Membrane

Several membrane protein complexes identified in this study have a function in molecule trafficking through the *C. jejuni* 81–176 membrane. These complexes play an important role in adaptation and virulence capabilities. For instance, the major *Campylobacter* porin PorA was extracted and identified in the complexes 3 and 13. This porin corresponding to MOMP, involved in the adaptation of *Campylobacter* to host environments (De et al., 2000; Zhang et al., 2000; Clark et al., 2007; Ferrara et al., 2016), is a multihomooligomeric porin with three PorA subunits (Zhang et al., 2000; Ferrara et al., 2016). This porin can be identified in three conformational forms including the folded monomer (35 kDa), the denatured monomer (40 to 48 kDa), and the native trimer (120 to 140 kDa) (Huyer et al., 1986; Zhang et al., 2000). In our gel, two complexes corresponding to native trimer MOMP at two different weights were identified. The complex 13 was estimated between 120–140 kDa and after subunit separation proteins corresponding to the folded monomer at 35 kDa and the denatured monomer between 40 and 48 kDa could be detected as previously described by Zhang et al. (2000). In the complex 3, the spot with apparent weight between 350 kDa and 400 kDa could correspond to the fusion of two trimeric MOMPs. The associated forms of MOMP in the complex 3 with likely a folded monomer, denatured monomer and a truncated form at 16 kDa was not previously described.

### Antibiotic Efflux Pumps

In this work, units of complexes corresponding to two efflux pumps CmeABC and MacAB were detected (complex 6 and 20). These two efflux pumps were known to play a role in antimicrobials resistance. The multidrug efflux pump CmeABC was more studied compared to the macrolide efflux pump MacABputC in *Campylobacter* (Lin et al., 2002, 2003; Akiba et al., 2006; Yan et al., 2006; Gibreel et al., 2007; Jeon and Zhang, 2009; Grinnage-Pulley and Zhang, 2015). In our study, the genetic and proteomic similarities between the constitutive proteins of these efflux pumps were highlighted by bioinformatics analyses. We were able to identify a potential third partner of the macrolide efflux pump, putMacC with a functional domain TolC similarly to CmeC. Subunits composing these two efflux pumps were mainly found in the proteobacteria phylum. Furthermore, the presence of CosR-binding box of the upstream sequences of *macA* was found in *C. jejuni* strains. However, this CosR-binding box could not be detected upstream *macA* in *C. coli* strains. Further biological analyses are required to explore the potential rule of CosR to regulate expression of transcripts of this macrolide efflux pump and to state its presumptive species specificity.

### New Membrane Protein Complexes

Two complexes (complex 1 and 14) were extracted and their subunits could be identified. The subunits of the complex 1, IlvC a ketol-acid reductoisomerase and Ftn a non-heme iron-containing ferritin were detected on the same horizontal line. IlvC is involved in L-isoleucine and L-valine biosynthesis (Pyndiah et al., 2007; Wu et al., 2016) while Ftn has a role in storing available cytosolic iron and to reduce cellular toxicity under conditions of intermittent or constant iron excess during infection (Wai et al., 1996). There are few information concerning these two proteins in *Campylobacter* and no interactions were reported. Complexes 10 and 14 are made of two isoforms of the methyl accepting chemotaxis protein CJJ_0180. This protein plays a role in chemotaxis and colonization of the gastrointestinal tract (Gonzalez et al., 1998; Zautner et al., 2012; Li et al., 2014; Chandrashekhar et al., 2015). However, weight of complexes 10 and 14 are different, indicating that these multihomooligomeric complex might be composed of different forms of the subunit as it was described for MOMP complex.

To conclude, this study is the first 2-D BN/SDS-PAGE method applied to identify membrane proteocomplexome of *Campylobacter jejuni*. Although not all the subunits of functional complexes in the membrane of *C. jejuni* could be detected, functional genomics analyses assisted us in reconstituting probable functional complexes. For instance, we were able to pinpoint a potential third partner in the macrolide efflux pump and raised hypothesis concerning its regulation by CosR. The 2-D BN/SDS-PAGE raised also limitations for studying bacterial proteocomplexomes. Assignment of spots to independent membrane protein complexes in low molecular weight areas is less easy. In certain cases, protein complexes were probably too weakly expressed as compared to others, or absent in optimal conditions. The tune up of DDM concentration, the conformation of complexes and their location, as full or part of the membrane, might contribute to the extraction of entire and stable complexes. Altogether, this study has allowed to better described the membrane proteocomplexome of *C. jejuni* providing new focus for further studies of protein complexes previously annotated with unknown functions.

## Data Availability Statement

The raw data supporting the conclusions of this article will be made available by the authors, without undue reservation, to any qualified researcher.

## Author Contributions

OT conceived the work and contributed to finishing the manuscript writing and preparing the figures. SS, LC, and AG performed the experiments. AG prepared the manuscript and the figures. OT, ED, LC, FB-H, and AM revised the manuscript. All authors contributed to the article and approved the submitted version.

## Conflict of Interest

The authors declare that the research was conducted in the absence of any commercial or financial relationships that could be construed as a potential conflict of interest.

## Footnotes

1 http://tree.bio.ed.ac.uk/software/figtree/

2 www.yeastrc.org/philius/pages/philius/runPhilius.jsp

3 http://weblogo.threeplusone.com

## Supplementary Material

Supplementary Figure 1 Homemade strips in 5 steps to separate the subunits of protein complexes in a reproducing manner. **(A)** Cut the strip with a clean glass plate following the well where the sample was deposited, equilibrate the gel lane in DTT and iodoacetamide buffers as indicated in materials and methods; **(B)** lay the gel strip on the wet plastic support using clean forceps; **(C)** Stick the plastic face of the strip on the glass behind; **(D)** Slide the strip until touching the precast polyacrylamide gel; **(E)** Fill the gap and overlaid the strip with low melting agarose using a sterile syringe.

Supplementary Figure 2 2D-BN-SDS-PAGE profiling before **(A)** and after optimization **(B)** of protein complexes and their subunit separation. The first dimension for complex separation was performed on 4 to 18% acrylamide gradient gels with 2% Dodecyl-β-D-Maltoside (DDM).

Supplementary Figure 3 2D-BN/SDS-PAGE western blot, using antibody anti-MOMP **(A)** and antibody anti-CadF **(B)**. The image in the middle corresponds to the identified proteins complexes before the Western-blot the black arrow indicate the identified proteins with anti-PorA antibody and the white arrow the identify protein with anti-CadF antibody on the 2D-BN/SDS-PAGE.

Supplementary Table 1 *Campylobacter* strains with complete genome sequencing used in this study.

Supplementary Table 2 Number of hits corresponding to the protein sequence of each three subunits of efflux pumps Cme and Mac of *C. jejuni* 81–176 in RefSeq_protein database. One hit is the result of sequence similarity using comparison tool Blastp with the parameters indicated in materials and methods section. The number of the different occurrences is defined by the threshold of word size.
