# Supplementary figures and images for: Membrane Proteocomplexome of Campylobacter jejuni Using 2-D Blue Native/SDS-PAGE Combined to Bioinformatics Analysis
Source: Front Microbiol. 2020 Nov 19;11:530906. doi: 10.3389/fmicb.2020.530906 (PMC7717971; doi:10.3389/fmicb.2020.530906)

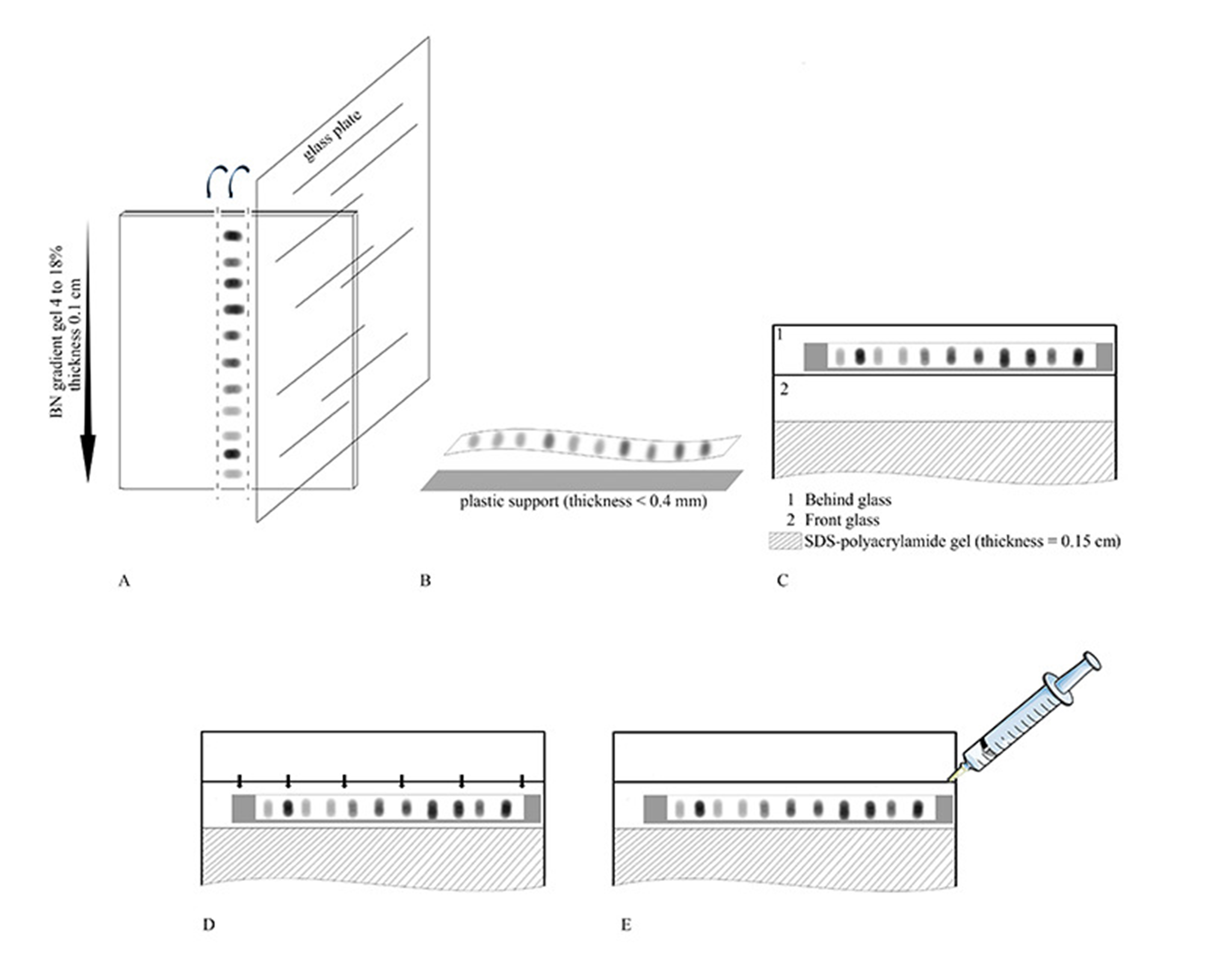

Supplement: Supplementary Figure 1 — Homemade strips in 5 steps to separate the subunits of protein complexes in a reproducing manner. (A) Cut the strip with a clean glass plate following the well where the sample was deposited, equilibrate the gel lane in DTT and iodoacetamide buffers as indicated in materials and methods; (B) lay the gel strip on the wet plastic support using clean forceps; (C) Stick the plastic face of the strip on the glass behind; (D) Slide the strip until touching the precast polyacrylamide gel; (E) Fill the gap and overlaid the strip with low melting agarose using a sterile syringe. [file Image_1.JPEG]

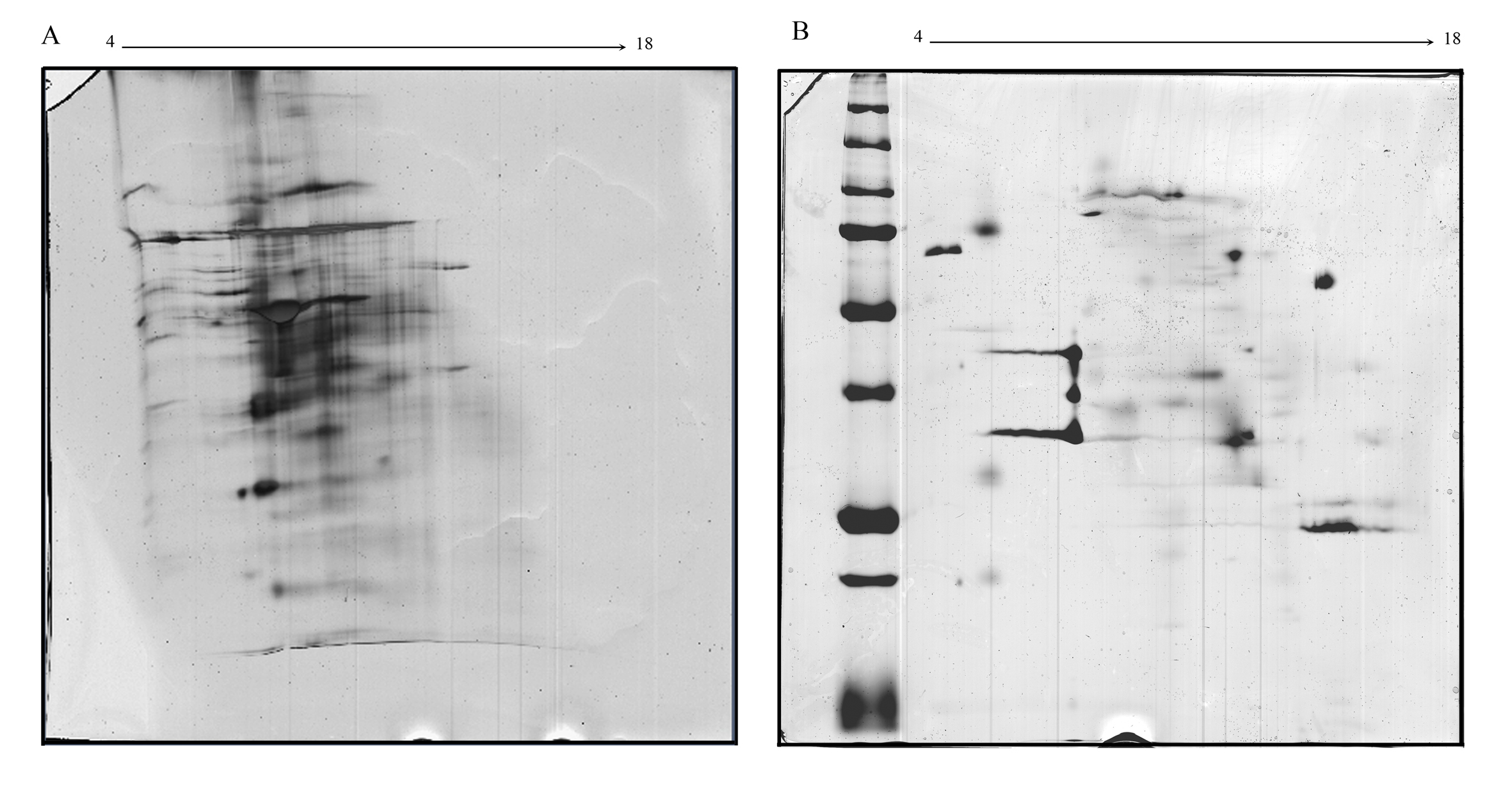

Supplement: Supplementary Figure 2 — 2D-BN-SDS-PAGE profiling before (A) and after optimization (B) of protein complexes and their subunit separation. The first dimension for complex separation was performed on 4 to 18% acrylamide gradient gels with 2% Dodecyl-β-D-Maltoside (DDM). [file Image_2.JPEG]

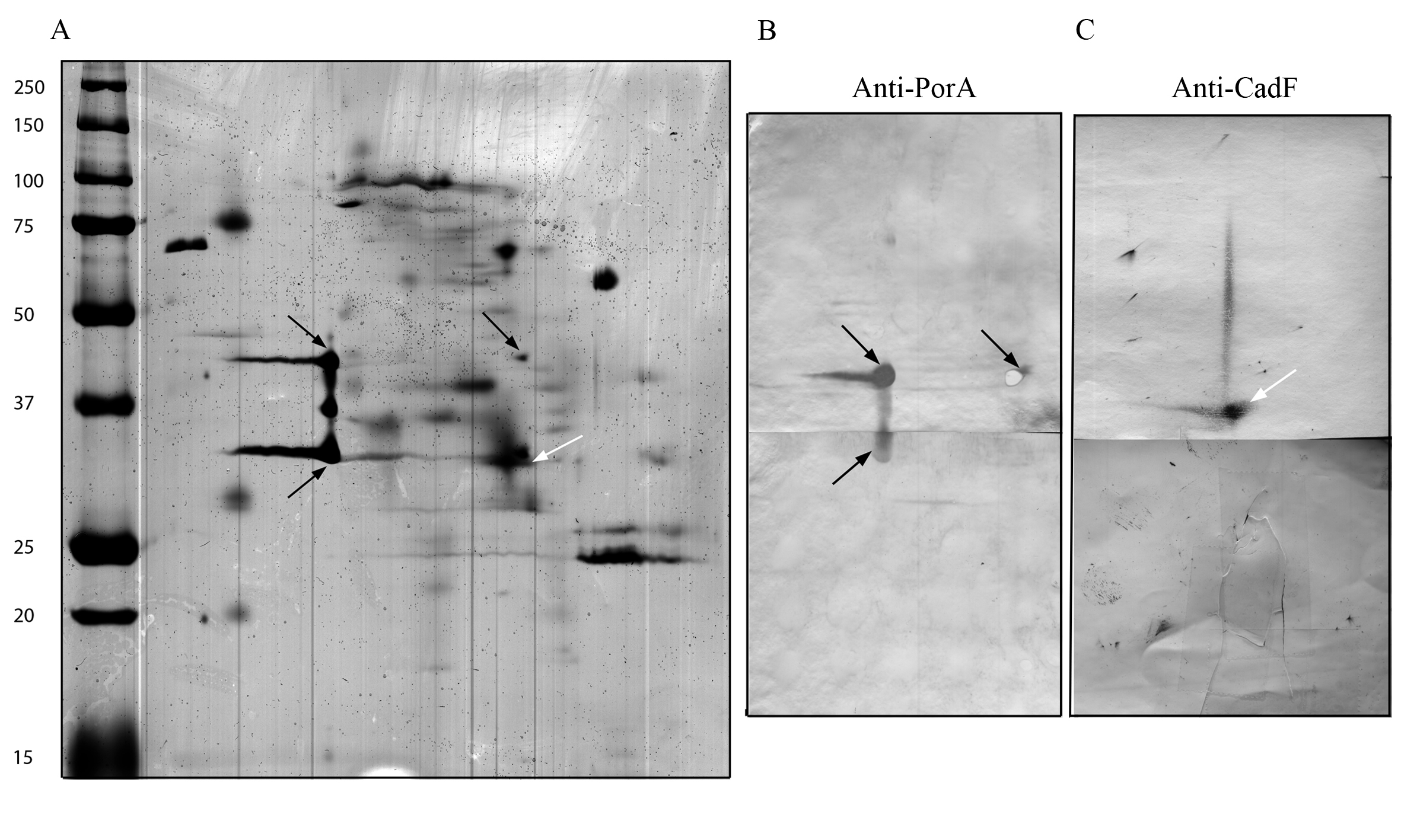

Supplement: Supplementary Figure 3 — 2D-BN/SDS-PAGE western blot, using antibody anti-MOMP (A) and antibody anti-CadF (B). The image in the middle corresponds to the identified proteins complexes before the Western-blot the black arrow indicate the identified proteins with anti-PorA antibody and the white arrow the identify protein with anti-CadF antibody on the 2D-BN/SDS-PAGE. [file Image_3.JPEG]
