# Supplementary material for: Membrane Proteocomplexome of Campylobacter jejuni Using 2-D Blue Native/SDS-PAGE Combined to Bioinformatics Analysis
Source: Front Microbiol. 2020 Nov 19;11:530906. doi: 10.3389/fmicb.2020.530906 (PMC7717971; doi:10.3389/fmicb.2020.530906)
Supplement: Supplementary Table 1 — Campylobacter strains with complete genome sequencing used in this study. [file Table_1.docx]

**Table S1**: *Campylobacter* strains with complete genome sequencing used in this study

|  | Genome sequence length (Mb) | GC% | Number of CDS | Accession |
| --- | --- | --- | --- | --- |
| *C. jejuni* 81-176 | 1.62 | 30.62 | 1671 | NC_008787 |
| *C. jejuni* NCTC 11168 | 1.64 | 30.55 | 1643 | NC_002163 |
| *C. jejuni* BF | 1.55 | 30.39 | 1709 | [FCEZ01000095](https://www.ncbi.nlm.nih.gov/nuccore/FCEZ01000095) |
| *C. jejuni* 00-2425 | 1.72 | 30.51 | 1754 | NC_022362 |
| *C. jejuni* 00-2426 | 1.62 | 30.43 | 1738 | NC_022352 |
| *C. jejuni* 00-2544 | 1.66 | 30.40 | 1799 | NC_022353 |
| *C. jejuni* 81116 | 1.63 | 30.54 | 1642 | NC_009839 |
| *C. jejuni* IA3902 | 1.64 | 30.57 | 1653 | NC_017279 |
| *C. jejuni* PT14 | 1.64 | 30.54 | 1680 | NC_018709 |
| *C. jejuni* 4031 | 1.67 | 30.47 | 1711 | NC_022529 |
| *C. jejuni* M1 | 1.62 | 30.6 | 1655 | NC_017280 |
| *C. jejuni* RM1221 | 1.78 | 30.31 | 1877 | NC_003912 |
| *C. jejuni* S3 | 1.68 | 30.49 | 1757 | NC_017281 |
| *C. jejuni* 00-2538 | 1.66 | 30.45 | 1787 | NC_022351 |
| *C. coli* RM4661 | 1.82 | 31.15 | 1876 | NZ_CP007181 |
| *C. coli* 15-537360 | 1.66 | 31.45 | 1663 | NC_022660 |
| *C. coli* 76339 | 1.58 | 31.88 | 1570 | NC_022132 |
| *C. coli* CVM N29710 | 1.67 | 31.44 | 1662 | NC_022347 |
| *C. coli* RM1875 | 1.81 | 31.17 | 1887 | NZ_CP007183 |
| *C. coli* RM5611 | 1.68 | 31.38 | 1691 | NZ_CP007179 |
