# Supplementary material for: Membrane Proteocomplexome of Campylobacter jejuni Using 2-D Blue Native/SDS-PAGE Combined to Bioinformatics Analysis
Source: Front Microbiol. 2020 Nov 19;11:530906. doi: 10.3389/fmicb.2020.530906 (PMC7717971; doi:10.3389/fmicb.2020.530906)
Supplement: Supplementary Table 2 — Number of hits corresponding to the protein sequence of each three subunits of efflux pumps Cme and Mac of C. jejuni 81–176 in RefSeq_protein database. One hit is the result of sequence similarity using comparison tool Blastp with the parameters indicated in materials and methods section. The number of the different occurrences is defined by the threshold of word size. [file Table_2.docx]

**Table S2:** Number of hits corresponding to the protein sequence of each three subunits of efflux pumps Cme and Mac of *C. jejuni* 81-176 in RefSeq_protein database. One hit is the result of sequence similarity using comparison tool Blastp with the parameters indicated in material and methods section. The number of the different occurrences is defined by the threshold of word size.

| Efflux pump | Cme | | | Mac | | |
| --- | --- | --- | --- | --- | --- | --- |
| Subunits | CmeA | CmeB | CmeC | MacA | MacB | MacC |
| Delta/epsilon bacteria | 1327 | 1484 | 463 | 1166 | 1208 | 1236 |
| Alphaproteobacteria | 253 | 603 | 14 | 27 | 28 | 1393 |
| Gammaproteobacteria | 1088 | 307 | 130 | 1508 | 1173 | 2 |
| Betaproteobacteria | 148 | 91 | 384 | 1593 | 1551 | 250 |
| Fusobacteriaceae |  |  |  | 35 | 27 | 32 |
| Others | 22 | 6 | 15 | 1 |  | 2 |
| Total number of hits | 2838 | 2491 | 1006 | 4330 | 3987 | 2915 |
| Number of different occurrences | 1143 | 980 | 397 | 1309 | 1089 | 886 |
